# The Portuguese version of the RhinoQOL Questionnaire: validation and clinical application^☆^^☆☆^

**DOI:** 10.1016/j.bjorl.2015.08.015

**Published:** 2015-10-21

**Authors:** Rui Cerejeira, Rafaela Veloso-Teles, Nuno Lousan, Carla Pinto Moura

**Affiliations:** a Department of Genetics, Faculty of Medicine, University of Porto, Portugal; b Department of Otorhinolaryngology, Tâmega e Sousa Hospital Center, Penafiel, Portugal; c Department of Otorhinolaryngology, Alto Ave Hospital Center, Guimarães, Portugal; d Department of Otorhinolaryngology, São João Hospital Center, Porto, Portugal

**Keywords:** Quality of life, Questionnaires, Sinusitis, Natural orifice endoscopic surgery, Qualidade de vida, Questionários, Sinusite, Cirurgia endoscópica por orifício natural

## Abstract

**Introduction:**

Rhinosinusitis constitutes an important health problem, with significant interference in personal, professional, and social functioning. This study presents the validation process of the Portuguese version of the RhinoQOL, to be used as a routine procedure in the assessment of patients with chronic rhinosinusitis.

**Objective:**

To demonstrate that the Portuguese version of the RhinoQOL is as valid as the English version to measure symptoms and health-related quality of life in chronic rhinosinusitis.

**Methods:**

The Portuguese version of the RhinoQOL was administered consecutively to 58 patients with chronic rhinosinusitis with and without nasal polyps, assessed for endoscopic sinus surgery. A follow-up survey was completed three months after surgery. Statistical analysis was performed to determine its psychometric properties.

**Results:**

Face and content validity were confirmed by similar internal consistency as the original questionnaire for each sub-scale, and strong correlation between individual items and total score. The questionnaire was easy and quick to administer (5.5 min). At three months, there was a significant decrease from baseline for all sub-scale scores, indicating clinical improvement, with an effect size considered as large.

**Conclusion:**

This study provides a questionnaire that is equivalent to the original English version, with good responsiveness to change, which can be especially valuable to measure the outcome of surgery.

## Resumo

**Introdução:**

A rinossinusite constitui um importante problema de saúde, com interferência significante na vida pessoal, e sócio-profissional dos pacientes. Este estudo apresenta o processo de validação da versão do RhinoQOL na língua portuguesa para ser usado em pacientes portadores de rinossinusite crônica.

**Objetivo:**

Demonstrar que a versão do RhinoQOL na língua portuguesa é tão válida quanto a versão inglesa na medição dos sintomas e qualidade de vida dos pacientes com rinossinusite crônica.

**Método:**

A versão em português do RhinoQOL foi aplicada consecutivamente a 58 pacientes com rinossinusite crônica, com e sem pólipos nasais, previamente à cirurgia endoscópica nasal, tendo sido reavaliados aos 3 meses de pós-operatório. Análise estatística foi realizada para determinar as suas propriedades psicométricas.

**Resultado:**

A validade de conteúdo foi confirmada por uma consistência interna similar à do questionário original, para cada sub-escala, e por uma forte correlação entre cada item e o *score* total. A aplicação do questionário foi fácil e rápida (5,5 min). Aos 3 meses, verificou-se uma redução significativa dos *scores* de todas as sub-escalas, indicando melhoria clínica, com um tamanho de efeito considerado grande.

**Conclusão:**

Este estudo fornece um questionário que é equivalente à versão original, com boa sensibilidade à mudança, o que pode ser especialmente útil na medição do impacto da cirurgia.

## Introduction

Rhinosinusitis, acute or chronic, with or without nasal polyps, constitutes an important health problem with significant impact on quality of life, interfering in personal, professional, and social functioning.^1^

Disease-specific instruments that measure symptoms and health-related quality of life (HRQL) have been developed to assess the impact of rhinosinusitis in individual patients, and to monitor the response to treatment. There are several questionnaires (Table 1) that subjectively access rhinosinusitis impact and associated incapacity.

**Table 1:** Questionnaires for assessment of quality of life in rhinosinusitis.

	Instrument	Items	Domains	Authors
RSOM-31	Rhinosinusitis Outcome Measurement	31	7	Piccirillo^2^
SNOT-16	Sinonasal Outcome Test	16	1	Anderson et al.^3^
SNOT-20	Sinonasal Outcome Test	20	1	Piccirillo et al.^4^
SNOT-22	Sinonasal Outcome Test	22	1	Hopkins et al.^5^, ^6^
RSDI	Rhinosinusitis Disability Index	30	3	Benninger, Senior^7^
RhinoQOL	Rhinosinusitis Quality of Life Survey Instrument	17	3	Atlas et al.^8^, ^9^

**Figure 1:**
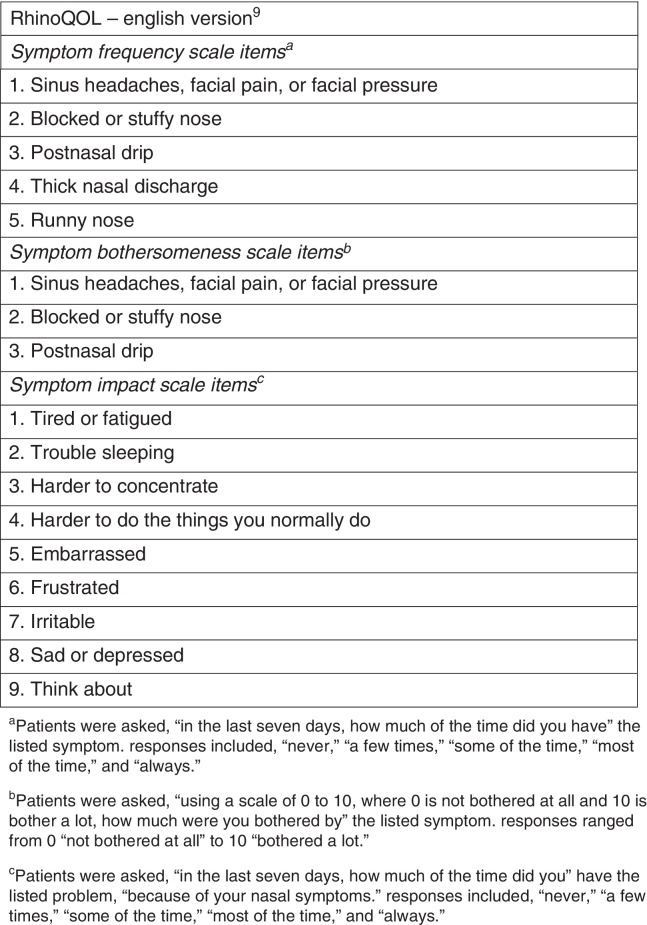
RhinoQOL – English version.

One of the major problems precluding the administration of these instruments in daily clinical practice is the lack of available time. The need for a brief and easy-to-use rhinosinusitis-specific questionnaire with strong psychometric characteristics resulted in the development of the RhinoQOL Survey Instrument^8^ (Fig. 1), that has been validated in acute^8^ and chronic^9^ rhinosinusitis patients treated both medically and surgically.

This assessment instrument, which has been already validated in French,^10^ consists of 17 items, divided in three domains addressing symptom frequency (five items), symptom bothersomeness (with answers ranging from 0, meaning “not bothered at all,” to 10, meaning “bothered a lot,” for each of its three items), and symptom impact (nine items). For the symptom frequency and impact questions, the patient has five possible responses: “never,” “a few times,” “some of the time,” “most of the time,” and “always.”

In a recent systematic review of the available HRQL questionnaires for Rhinosinusitis, the RhinoQOL was one of only two (the other being the RSOM-31) that met the authors’ criteria for discriminant validity and responsiveness.^11^

This article presents the translation to Portuguese and validation procedure of the RhinoQOL to allow its use in assessment of chronic rhinosinusitis (CRS) in Portuguese-speaking patients.

## Methods

The present study was approved by the Ethical Committee of a level 1 hospital (decision 12/2012) and was performed according to the principles of the Declaration of Helsinki.

### Adaptation to Portuguese

**Figure 2:**
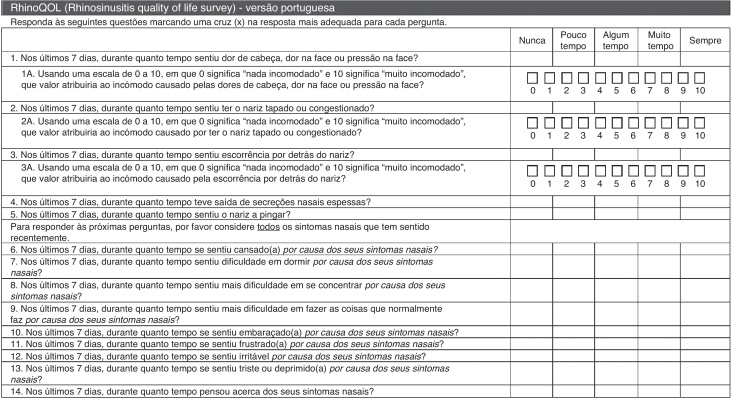
RhinoQOL – Portuguese version.

The RhinoQOL questionnaire was obtained from the original article by Atlas et al.^9^ (Fig. 1). A double translation of the English questionnaire into Portuguese was made by two bilingual physicians, followed by a retrotranslation into English. The final form of the validated Portuguese version of the RhinoQOL (RhinoQOL-pv) is attached to this article (Fig. 2).

### Sample and procedures

The study was conducted in the Otolaryngology Department on patients submitted to surgery from December 1, 2012 to July 31, 2013. The RhinoQOL-pv was administered, during the consultation, consecutively to the first 58 patients with CRS with nasal polyps (CRSwNP) and without nasal polyps (CRSsNP) assessed pre-operatively for endoscopic sinus surgery (ESS), which was always performed by the same surgical team, using the principles of functional endoscopic surgery first described by Messerklinger.^12^ The sample size (*n* = 58) was determined based on the sample size of the original study by Atlas et al.^9^ (*n* = 50), in which it was possible to detect clinically significant differences. All persons provided informed consent prior to their inclusion in the study. Eligibility criteria included age 18 or older and ability to speak and read Portuguese. Administration time was measured (in minutes). Patients were asked about the clearness of questions (“Did you experience difficulties in interpreting any of the items of the questionnaire?”) and the duration of the assessment (“Did you find the questionnaire too long to complete?”) with a yes/no answer option. Patients completed a follow-up survey three months after surgery.

### Statistical procedure

Data collected in the assessment were introduced and processed by the statistical software IBM™ SPSS™ Statistics v. 21.

Descriptive statistics of demographic and clinical data of the sample were calculated (age, sex, presence of nasal polyps, and Lund–MacKay score^13^ in pre-operative computed tomography scans).

As in the original study of the RhinoQOL,^9^ the psychometric evaluation was performed separately for symptom frequency, bothersomeness, and impact scales. Scores for the symptom frequency and impact scales ranged from 1 (“never”) to 5 (“always”). For the bothersomeness scale, scores ranged from 0 to 10, in accordance with the questions possible answers.

Internal consistency reliability was assessed using Cronbach's alpha. For comparison of means between two groups, Student's *t*-test was used. Correlation between Lund–MacKay score and RhinoQOL sub-scale scores was tested with Pearson's coefficient for quantitative variables; correlation between each individual item and total RhinoQOL-pv sub-scale scores was tested with Spearman's coefficient.

The change in scores between baseline and three month follow-up was assessed using Student's *t*-test for paired samples. Responsiveness was also assessed by measuring the magnitude of the effect, which is the mean value of variation of the scores divided by the standard deviation of the initial values. By convention, an effect magnitude between 0.2 and 0.5 is considered a “mild” improvement; between 0.5 and 0.8, “moderate” improvement; and greater than 0.8, a “large” improvement in quality of life.^6^

## Results

The sample consisted of 58 patients, 30 men and 28 women. Mean age was 48.48 ± 11.703 years (range, 25–69 years). Thirty-eight patients suffered from CRSwNP. Cases are distributed according to Table 2.

**Table 2:** Distribution of patients according to sex and type of CRS.

	CRSwNP	CRSsNP	Total
Male	20	10	30
Female	18	10	28

CRSwNP, chronic rhinosinusitis with nasal polyps; CRSsNP, chronic rhinosinusitis without nasal polyps.

Five patients (8.6%) answered “yes” to the question about difficulty of interpretation of the items. On average, the questionnaire took 5.53 ± 1.127 min (range, 4–8 min) to complete. Fifty-six patients (96.6%) reported that the RhinoQOL-pv was not too long to complete.

Internal consistency (Cronbach's *α*) was 0.77, 0.88, and 0.56 for the frequency, impact, and bothersomeness scores, respectively.

As depicted in Table 3, male sex was associated with lower total score in the Symptom Impact Scale than female sex (Student's *t*-test significance = 0.018). Moreover, CRSwNP was associated with higher frequency scale scores than CRSsNP (Student's *t*-test significance = 0.009) (Table 4).

**Table 3:** Total score by sex for each sub-scale.

Sex	*n*	Frequency scale		Impact scale		Bothersomeness scale	
		Mean	SD	Mean	SD	Mean	SD
Male	30	14.71	5.01	17.60	4.66	13.07	6.31
Female	28	12.93	4.24	22.71	9.97	15.46	6.41
Significance (*α* = 0.05)		0.149		0.018		0.157

**Table 4:** Total score by type of CRS for each sub-scale.

	*n*	Frequency scale		Impact scale		Bothersomeness scale	
		Mean	SD	Mean	SD	Mean	SD
CRSwNP	38	14.79	5.10	19.37	6.72	14.95	7.15
CRSsNP	20	11.90	3.02	21.40	10.18	12.85	4.56
Significance (*α* = 0.05)		0.009		0.428		0.180	

CRSwNP, chronic rhinosinusitis with nasal polyps; CRSsNP, chronic rhinosinusitis without nasal polyps.

Mean Lund–Mackay global score was 13.52 ± 4.94 (range, 4–23). Patients with CRSwNP presented with higher Lund–Mackay scores compared to patients with CRSsNP (Table 5). A modest positive correlation (*r* = 0.380) was obtained between the symptom frequency sub-scale of the RhinoQOL-pv and the total Lund–MacKay score (*p* = 0.003). No association with other sub-scales was identified.

**Table 5:** Lund–Mackay total score by type of CRS.

	*n*	Mean	SD	Significance (*α* = 0.05)
CRSwNP	38	15.89	3.965	<0.001
CRSsNP	20	9.00	3.112	

CRSwNP, chronic rhinosinusitis with nasal polyps; CRSsNP, chronic rhinosinusitis without nasal polyps.

Scores of every individual item of the RhinoQOL-pv correlated significantly with total score of each sub-scale, as shown in Table 6.

**Table 6:** Spearman coefficient and significance (two-tailed) for correlation between individual items and total score of each sub-scale of the RhinoQOL-pv (*α* = 0.05).

Sub-scale of RhinoQOL	Item	Spearman	Significance
Frequency	1	0.489	<0.001
	2	0.605	<0.001
	3	0.907	<0.001
	4	0.838	<0.001
	5	0.763	<0.001

Impact	1	0.739	<0.001
	2	0.689	<0.001
	3	0.676	<0.001
	4	0.643	<0.001
	5	0.390	0.002
	6	0.844	<0.001
	7	0.834	<0.001
	8	0.663	<0.001
	9	0.653	<0.001

Bothersomeness	1	0.675	<0.001
	2	0.670	<0.001
	3	0.829	<0.001

Scores on each sub-scale at baseline and three month follow-up are shown in Table 7. There was a significant decrease from baseline for all sub-scale scores, indicating clinical improvement. At three months, the effect size in all patients was considered large for all sub-scales (Table 8).

**Table 7:** Responsiveness: scores at baseline and at three-month postoperative follow-up (*α* = 0.05).

RhinoQOL sub-scales	Baseline			Three-month follow-up			*p*-Value
	*n*	Mean	SD	*n*	Mean	SD	
Frequency	58	13.79	4.67	58	8.79	2.19	<0.001
Impact	58	20.07	8.05	58	12.95	4.35	<0.001
Bothersomeness	58	14.22	6.42	58	5.31	3.81	<0.001

**Table 8:** Responsiveness: magnitude of effect – total sample and by type of CRS.

RhinoQOL sub-scales	(Mean_baseline_ − Mean_3month_)/SD_baseline_			Improvement in QOL
	Total	CRSwNP	CRSsNP	
Frequency	1.07	1.19	0.99	Large
Impact	0.88	0.95	0.83	Large
Bothersomeness	1.39	1.31	1.77	Large

QOL, quality of life; CRSwNP, chronic rhinosinusitis with nasal polyps; CRSsNP, chronic rhinosinusitis without nasal polyps.

## Discussion

Clinical evaluation of CRS, with and without nasal polyps, is essentially focused on patient complaints of nasal blockage and discharge, facial pain/pressure, and reduction or loss of smell, with objective evidence of disease demonstrated by endoscopy or CT. There is a wide range of additional assessment tools; most of them are mainly used for research purposes.^1^

Questionnaires that integrate patient reported symptoms and their impact on HRQL are increasingly useful, and their use is now becoming a routine procedure in the assessment of patients with rhinosinusitis. To date, the only validated Portuguese version of such questionnaires published in international literature is the translated version of the Sinonasal Outcome Test (SNOT-22).^14^

The RhinoQOL is a new questionnaire that showed validity and responsiveness comparable to the RSOM-31 and SNOT instruments, and demonstrated excellent responsiveness to change over time associated with surgery.^9^ The RhinoQOL-pv proved to be an adequate translation of the English version as shown by similar internal consistency as the original questionnaire for each sub-scale (Table 9), and strong correlation between individual items and total score. In addition, patients to whom the RhinoQOL-pv was administered did not experience major difficulties.

**Table 9:** Internal consistency reliability – Cronbach's *α*.

	RhinoQOL (original)^9^	RhinoQOL-pv
Cronbach's *α* [Frequency]	0.68	0.77
Cronbach's *α* [Impact]	0.89	0.88
Cronbach's *α* [Bothersomeness]	0.57	0.56

Of the variables tested, sex was associated with differences in the total score of the impact sub-scale of the RhinoQOL-pv, with men presenting statistically significant lower scores. This finding raises the question whether levels of reported distress may reflect higher levels of comorbid anxiety disorders known to be more prevalent in women.^15^ Also, patients with CRSwNP showed significant higher frequency sub-scale scores compared to patients without nasal polyps, but without any statistically significant differences in bothersomeness and impact sub-scale scores, suggesting that tolerability to disease is not different in the two conditions.

Although many studies have demonstrated lack of correlation between patient rated measures of symptom severity in CRS and objective measures,^1^ a significant yet modest positive correlation was verified between the symptom frequency sub-scale and the radiological Lund–Mackay scoring system.

Score changes from baseline to the three-month postoperative follow-up showed that the RhinoQOL-pv is a useful tool in the assessment of ESS results, with good responsiveness to change. In fact, interpretation of raw data from the RhinoQOL-pv in a single (static) moment of time may prove to be difficult, as results are not intuitively inferred; however, interpretation of differences before and after surgical treatment appeared to be much easier to perceive.

## Conclusion

The RhinoQOL-pv can be used for Portuguese speaking patients with CRS as an equivalent of the original English version, because they have similar face and content validity. This study provides an instrument with good responsiveness to change, which could be especially valuable to measure the outcome of ESS.

## Conflicts of interest

The authors declare no conflicts of interest.
